# Contrasting Epidemiology of Cholera in Bangladesh and Africa

**DOI:** 10.1093/infdis/jiab440

**Published:** 2021-09-22

**Authors:** David A Sack, Amanda K Debes, Jerome Ateudjieu, Godfrey Bwire, Mohammad Ali, Moise Chi Ngwa, John Mwaba, Roma Chilengi, Christopher C Orach, Waqo Boru, Ahmed Abade Mohamed, Malathi Ram, Christine Marie George, O Colin Stine

**Affiliations:** 1 Department of International Health, Johns Hopkins Bloomberg School of Public Health, Baltimore, Maryland, USA; 2 Meilleur Acces aux Soins de Sante, and Department of Public Health, Faculty of Medicine and Pharmaceutical Sciences, University of Dschang, and Clinical Research Unit, Division of Health Operations Research, Cameroon Ministry of Public Health, Yaoundé, Cameroon; 3 Department of Integrated Epidemiology, Surveillance, and Public Health Emergencies, Ministry of Health, Kampala, Uganda; 4 Centre for Infectious Disease Research in Zambia, Lusaka, Zambia; 5 Department of Community Health and Behavioural Sciences, Makerere University School of Public Health, Kampala, Uganda; 6 Ministry of Health and Field Epidemiology and Laboratory Training Program, Nairobi, Kenya; 7 Tanzania Field Epidemiology and Laboratory Training Program, Dar-es-Salaam, Tanzania; 8 Department of Epidemiology and Public Health, School of Medicine, University of Maryland, Baltimore, Maryland, USA

**Keywords:** cholera, Bangladesh, Africa, epidemiology, Cameroon, Uganda, refugee, emergencies GTFCC, roadmap

## Abstract

In Bangladesh and West Bengal cholera is seasonal, transmission occurs consistently annually. By contrast, in most African countries, cholera has inconsistent seasonal patterns and long periods without obvious transmission. Transmission patterns in Africa occur during intermittent outbreaks followed by elimination of that genetic lineage. Later another outbreak may occur because of reintroduction of new or evolved lineages from adjacent areas, often by human travelers. These then subsequently undergo subsequent elimination.

The frequent elimination and reintroduction has several implications when planning for cholera’s elimination including: a) reconsidering concepts of definition of elimination, b) stress on rapid detection and response to outbreaks, c) more effective use of oral cholera vaccine and WASH, d) need to readjust estimates of disease burden for Africa, e) re-examination of water as a reservoir for maintaining endemicity in Africa. This paper reviews major features of cholera’s epidemiology in African countries which appear different from the Ganges Delta.

Cholera continues to affect many countries around the world, having spread from its homeland in the Indian subcontinent to other countries in Asia, Africa, and Hispaniola. The disease, caused by intestinal infection with *Vibrio cholerae,* serotype O1 or O139, is characterized by acute watery diarrhea. In severe cases, this leads to rapidly progressing severe dehydration and, if not treated promptly, could lead to death within a few hours. Two recent estimates of cholera disease burden concluded that between 95 000 and 107 000 deaths result from the 2.86 to 2.88 million episodes of cholera annually [1, 2]. The disease is transmitted by the fecal-oral route and is more common in areas with poor water and sanitation. Much of the research on cholera has taken place in the Ganges Delta where the disease occurs consistently, including in Bangladesh at the International Centre for Diarrhoeal Disease Research, Bangladesh (icddr,b) and in West Bengal at the National Institute of Cholera and Enteric Diseases. Thus, many of the concepts of its epidemiology, transmission, methods for treatment and prevention, and its persistence in environmental reservoirs were developed from this research in the Ganges Delta.

Many cases occur in Asia, though they are rarely reported to the World Health Organization (WHO). Since 1970, most of the cases that are reported are from Africa [3]. With an emphasis on cholera control in Africa, increasing efforts are being carried out in Africa to understand the true burden, transmission patterns, and methods to document impact of vaccine and water-sanitation-hygiene (WASH) interventions. The studies in Africa benefit from the methods and concepts developed in Asia; however, differences in disease patterns suggest that some of these concepts and methods from Asia need to be reevaluated when applied to most countries in Africa.

## CHOLERA EPIDEMIOLOGY IN THE GANGES DELTA

**Figure 1.**
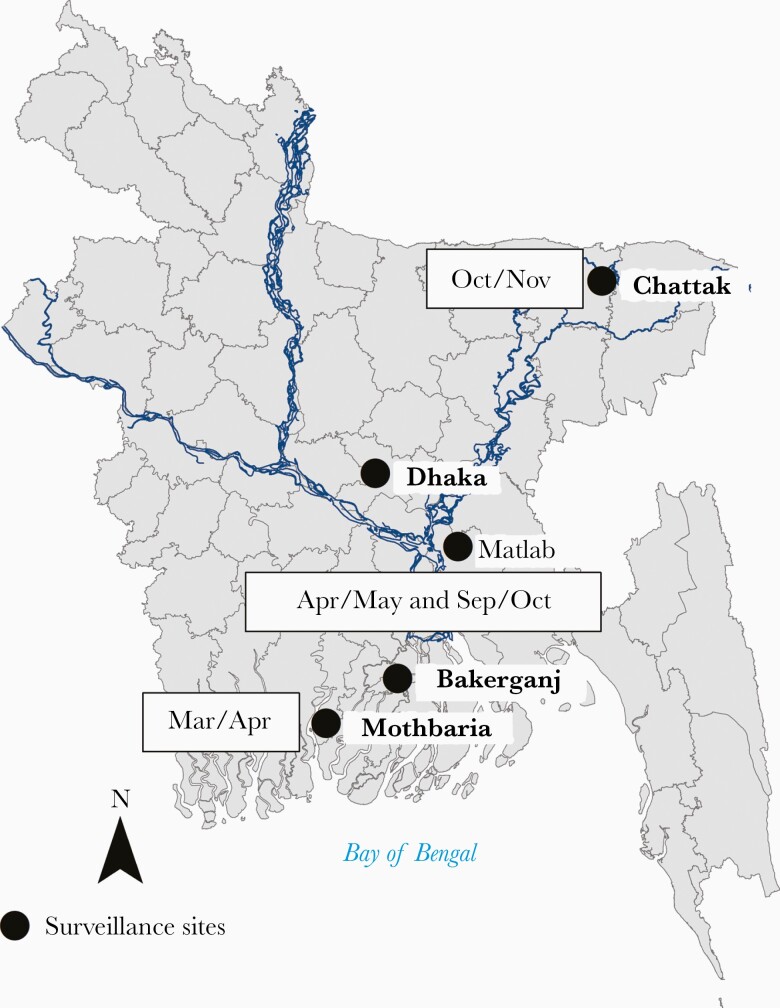
Identification of the months with high rate of cholera in Bangladesh (source of map https://gadm.org/maps/BGD.html) [6].

Cholera is endemic in the Ganges Delta. The WHO defines cholera as being endemic when it occurs in country during 3 of 5 years [4]. In Bangladesh cases occur throughout the year, every year. Rates of cholera vary during the season, but cases are documented every month of the year at the icddr,b hospital in Dhaka [5]. As illustrated in Figure 1, in Dhaka, the high season occurs before and after the monsoon (June to August) and numbers decrease during the cooler season (January to February) and during the monsoon. In the northern part of the country, the high season is October to November and in the southern part, the season is March to April [6]. Thus, even though the country is geographically small, being only 500 miles from north to south, these distinct seasonal differences are key features of the disease. Epidemiologists often refer to cholera in terms of outbreaks, but for Bangladesh the outbreak continues indefinitely without an end.

As is typical of other endemic diseases, young children have the highest rates of cholera [7]. The decreasing rates of disease with age are thought related to acquired immunity, and this is supported by the increasingly elevated vibriocidal antibody titers by age in the population [8, 9]. While rates are highest in young children, in fact, more older children and adults are affected because these older age groups make up a larger proportion of the population.

In Bangladesh, sentinel surveillance conducted in preselected facilities in different parts of the country is a useful method to characterize cholera seasonality and disease burden. Two key sites for such surveillance are the hospitals of the icddr,b in urban Dhaka and rural Matlab [5]. Other sentinel sites were established in other parts of the country based on convenience, logistical considerations, and specific scientific questions of interest [6, 7, 10].

With knowledge of the catchment population around the sentinel site, one can determine rates of disease from season to season and year to year. One such area used to determine precise rates of cholera is the demographically and geographically defined Matlab area [11]. With a population of more than 200 000, the etiology of diarrhea of those seeking care at the icddr,b hospital in Matlab is confirmed by microbial culture. Until recently, rates of cholera regularly exceeded 1 per 1000 every year. The detailed information on cholera cases by time and place has allowed for many epidemiological studies, vaccine field trials, and water interventions [12, 13]. Vaccine trials were also possible by creating demographically defined urban areas in Dhaka and Kolkata where there have been high and consistent rates of disease [14–16].

The consistently high rates of cholera also make it possible to understand transmission through family studies in which household members of cholera cases are studied prospectively to detect secondary cases and asymptomatic infections and to determine risk factors for these symptomatic and asymptomatic infections. These studies show that only about 20% of infected persons in Bangladesh develop severe disease [17–19]. They also provide evidence that markers of immunity [20] are protective and that intensive WASH interventions can prevent transmission within the household [19]. Additional studies are being planned to determine if an intensive, vaccine and/or WASH intervention will prevent transmission to the immediate neighbor households.

*Vibrio* species, including *V. cholerae*, naturally inhabit environmental waters [21] and identification of a persistent, viable but nonculturable (VBNC) form of *V. cholerae* O1 provides a hypothesis for an environmental reservoir for cholera [22]. Their presence in the environment suggested that, under certain climatic or other environmental conditions, the VBNC *V. cholerae* might infect people, leading to human disease and onward transmission. Because VBNC *V. cholerae* cannot be cultured, it is difficult to establish its true role as a reservoir for initiating clinical disease and outbreaks.

Environmental studies also showed that *V. cholerae* O1 become associated with plankton on which they may persist for a prolonged period [23]. The association with plankton suggested that people might ingest a large inoculum of bacteria if they consumed water with *Vibrio-*contaminated plankton, and that filtering drinking water might reduce the inoculum and reduce risk of cholera. In fact, cholera rates were decreased by about 50% in Matlab communities when cloth sari material was used to filter water [13].

The role of water in cholera transmission was also shown by detecting *V. cholerae* in household water, including source water being collected for the household. Often, but not always, the genotypes of the *V. cholerae* in the water were like those isolated from the stools of cholera patients, suggesting that the contaminated water was, in fact, the source of the infection. When the genotype of the isolates in source water from multiple households is the same as the outbreak isolates, it seems likely the source water initiated the outbreak. However, when the genotype of the *V. cholerae* in the water was not the same as the patient stool specimens, then the water could not be the source [24].

Persons infected with *V. cholerae* O1 develop an immune response, which protects them for several years from subsequent disease [25, 26]. Similarly, oral cholera vaccine (OCV) stimulates immunity for 3 to 5 years [16, 27]. Because both natural infections and vaccine stimulate immune protection, persons who were vaccinated and then experience natural exposure are likely to develop a boost in their protective immunity. Similarly, persons who were previously naturally exposed will develop an enhanced immune response if they are vaccinated. The resulting effectiveness of vaccine is likely the result of this interaction between vaccination and natural exposure.

Other biological factors have also been found to affect the risk for cholera. *Vibrio* is rapidly killed when exposed to gastric acid and persons with hypochlorhydria have increased risk [28, 29]. Persons with blood group O have higher rates of severe cholera compared to persons with other blood groups [30–33] and Lewis blood group antigen may also affect susceptibility to severe cholera [34]. Key features of cholera’s epidemiology in Bangladesh are summarized in the middle column of Table 1.

**Table 1.** Comparison of Cholera in the Ganges Delta and Many Countries in Africa

Feature	Ganges Delta	Africa
Endemicity	Cholera cases are reported every year, throughout the year	Sporadic outbreaks
Seasonality	Different regions within the country have peak rates depending on season The seasonal peaks are consistent, year to year	Some countries have a strong seasonality (eg, Burundi), but outbreaks may occur during different seasons
Geographic consistency	The same areas are affected from year to year	Hotspots identified but variable from year to year for most countries
Outbreaks	In Bangladesh, cases never stop; thus, it is difficult to define the end of an outbreak	Cholera occurs during well-defined outbreaks with a clear start and end
Risk by age group	The highest rates occur among young children aged 2–5 y	Similar rates across the age groups
Risk by sex	Higher number of cases in young boys compared to girls Higher numbers in women aged 15–45 y compared to men	Similar to Asia, but needs more study
Asymptomatic infections	Most infections are asymptomatic or mildly symptomatic	Needs further study
Biological susceptibility to severe disease	Persons with hypochlorhydria, with blood group O and possibly Lewis blood group Le(a+ b−) have higher rates	Not known
Methods to monitor disease burden	Sentinel surveillance at preselected sites is efficient when monitoring rates of disease and disease burden	Sentinel surveillance has limited value, but broad-based detection with rapid reporting is needed
Relation between endemic disease and vaccine effectiveness	Preexisting immunity affects vaccine response Vaccine response affects response to future natural exposures Protection results from combination of vaccine and natural exposure	Vaccine stimulates immune protection, but natural exposure has limited effect
Detection of *Vibrio cholerae* in environmental water	*V. cholerae* can be identified frequently in ponds and rivers as well as drinking water at the source and in the household	*V. cholerae* is rarely detected More studies are needed to determine optimal methods
Viable but not culturable (VBNC) *V. cholerae*	VBNC forms of *V. cholerae* can be identified throughout the year in pond water	Not yet studied
Genetic characteristics of *V. cholerae*	Multiple genotypes circulating in the country	A single genotype, or few types spread through an area

## STUDIES IN AFRICAN COUNTRIES WITH CHOLERA

After studying cholera in Bangladesh for many years, our group at Johns Hopkins University initiated studies in several African countries, starting in Cameroon. Cameroon was identified as a cholera hotspot in Africa, especially the Far North and Littoral Regions of the country [35, 36]. These areas experienced a very large cholera outbreak in 2010–2011, during which 33 192 cases with 1440 deaths (case fatality rate = 4.3%) were reported to WHO. The Lake Chad area seemed to be an ideal site to study the clinical, epidemiological, and ecological aspects of cholera because Lake Chad is a large shallow lake where people live in close association with the lake, many of whom subsist on fishing.

Based on methods for studying cholera’s epidemiology in Bangladesh, we established 9 sentinel surveillance sites in hospitals and clinics near Lake Chad although the numbers of reported cases had decreased since the major outbreak in 2011. When designing the surveillance, we made several assumptions based on findings from Bangladesh.

First, because the area was already defined as a major hotspot in Africa, we assumed that cholera was endemic and that cases would be identified readily. Second, as described in the *Cholera Fact Sheet* from the WHO [4], cholera surveillance methods are insensitive in Africa but we assumed that an intensive surveillance system would detect an accurate count, including cases that might not be recognized by a routine system. Third, we assumed that some cases, as in Bangladesh, would be mild or asymptomatic, requiring inclusion of mild as well as severe cases in the surveillance. Fourth, we assumed that cholera may be seasonal, requiring an extended period of surveillance, at least 3 years, to fully understand its seasonality. Fifth, we expected that a cholera outbreak may start with a few mild cases with higher numbers subsequently. Detection of these mild, early cases might provide an early warning for an impending outbreak. Finally, we hoped that by testing environment water for cholera, one might find an early warning for an outbreak.

Although these sentinel sites had reported many cholera cases previously, and these sites did report many cases of diarrhea during the intensive surveillance, both mild and severe, none were confirmed as cholera until an outbreak occurred in the Far North Region in 2014 [37]. The other diarrhea cases, except during the outbreak, had other etiologies, but were not cholera. Thus, this intensive surveillance for 3 years was not able to confirm the presence of any cases of cholera, which may have been occurring at a low rate or with mild symptoms, except cases that occurred during the 2014 outbreak, and this outbreak appeared to spread from nearby Nigeria. Also, monthly water samples from 30 various water collection sites in Cameroon did not (except for 1) detect any *V. cholerae* O1. The water sampling did detect many (approximately 20%) specimens positive for *V. cholerae* non-O1. The 1 positive water sample for *V. cholerae* O1 was collected during an outbreak from a well for drinking water on an island in Lake Chad. After detecting this positive sample using a rapid diagnostic test (RDT) method [38], the contaminated well was closed the next day and case numbers decreased on the island. A similar study of multiple environmental water sources in Uganda also found many samples positive for non-O1 *V. cholerae* O1 but no toxigenic *V. cholerae* O1 [39].

**Figure 2.**
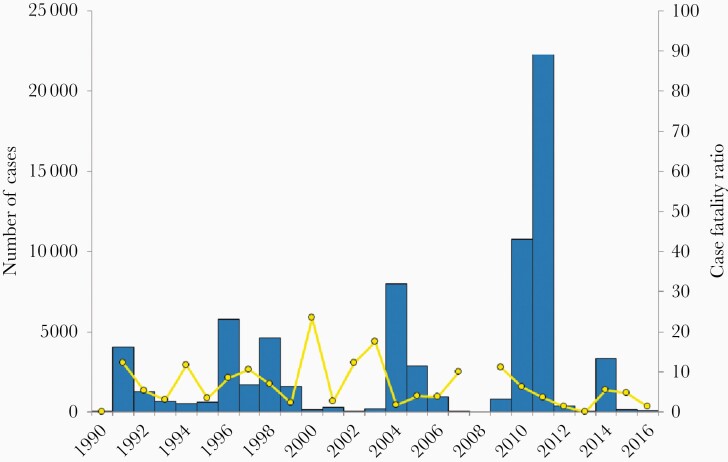
Yearly number of cholera cases (columns) and case fatality ratio (circles) in Cameroon 1990–2016 (data from [41]).

The national cholera surveillance for Cameroon [40] shows that cases are reported during many years, but in some years the numbers were either zero or very low, suggesting that cholera was present in Cameroon intermittently but not continuously (Figure 2) [41]. A cholera distribution pattern, different from that of Bangladesh, was also observed in a multicountry study in 7 enhanced surveillance zones and 4 outbreak sites in Togo, Democratic Republic of the Congo (DRC), Guinea, Uganda, Mozambique, and Cote d’Ivoire [42] and from the national reports from other African countries, including Guinea-Bissau, Ghana, and Zambia [43]. Cholera was reported frequently but years with many cases were interspersed by other years with no or few cases.

Examining data at a subnational level, countries where cholera is deemed to be endemic do report cholera within their national borders often, but the cases do not necessarily occur in the same districts year to year, as they do in Bangladesh, and countries that report cholera annually identify cases in different districts from year to year. Hotspot districts, where cases are seen more frequently, can be identified but even these hotspot districts do not report cholera every year and often have no cases for several consecutive years [36, 44]. Between these outbreaks, even within hotspot districts, cholera appears to have disappeared.

### Most Outbreaks in Africa Are Short

In contrast to the cholera seasons in Bangladesh, which persist indefinitely, most outbreaks (with a few exceptions) in Africa are relatively short. An example is the outbreak in Uganda that lasted 10 weeks [45]. No cases were seen during the other weeks in the year. Similar short outbreaks are documented in Tanzania (unpublished data) and Burundi [46].

### Implications for Surveillance and Disease Burden Estimates

The sentinel surveillance model, which detects cases at preselected sites, adapted from Bangladesh, was found to be insufficient, as illustrated in Cameroon. Although we expected to find cases in a defined hotspot, in fact, cholera was just as likely to appear in a different area that was not selected. Outbreaks seem to occur sporadically and not in specific, predetermined sites. Thus, an attempt to determine rates of disease and disease burden through sentinel surveillance was not helpful in Africa. Rather, a surveillance system needs to be alert for cholera outbreaks whenever and wherever they may occur and not be limited to a specific location.

When estimating disease burden, the consistent pattern seen in Bangladesh, where one could estimate an average incidence of disease, was not seen in Africa. Numbers of cases varied widely from year to year and an average or a median rate varied considerably from the observed rates. This calls into question the current estimates of about 2.86 or 2.88 million cases, which assumed rates of cholera between 2 and 4 per 1000 for sub-Saharan Africa [1]. If these relatively high rates occur only from time to time, or if the high rates apply to only some limited areas of the country, the average rate is actually much lower. Similarly, if cholera occurs during defined outbreaks and not as an endemic infection, the numbers will also be much lower. Although surveillance systems in African countries may underestimate the true number of cases during an outbreak, the severity of the undercount is likely much less than has been assumed and this underestimate is partially compensated by overcounting diarrhea cases that are not cholera. If most cases only occur during outbreaks, the actual number of cases occurring in Africa is likely to be much lower than previously estimated.

### Cholera Elimination as Applied to Africa

One of the goals of the Global Task Force on Cholera Control (GTFCC) is to eliminate cholera from *>* 20 countries by the year 2030 [47]. Elimination means no cases in an area for at least 3 years. While only a few African countries would qualify as having eliminated cholera, in fact, many districts within the countries would qualify. The pattern of cholera in most African districts (subnational areas) is one of repeated elimination. Countries are considered cholera endemic not because of continued transmission, as in Bangladesh, but rather because cholera frequently occurs in some district(s) within the country. We hypothesize that the pattern of cholera in most African countries (not including DRC and perhaps Mozambique) is for repeated elimination within a district but then with subsequent reintroduction. This suggests that cholera elimination within a country should focus on eliminating cholera from each district and monitor the number of districts where elimination has been achieved. The goal of the national control programs would be to prevent cholera reintroduction into these districts and to gauge success by maintaining district-level elimination.

Recent studies using whole-genome sequencing (WGS) on transmission of cholera to Africa from South Asia reinforces this concept that cholera is repeatedly introduced into Africa. As shown by Weill et al [48], 12 transmission events took place between 1970 and 2014. The *V. cholerae* genetic lineages transmitted to Africa were termed T1 to T12. Later, T13 was identified in Uganda [49], Zambia [50], and Yemen [51]. Interestingly, most of the earlier genetic lineages are no longer seen in Africa, so the pattern seems to be one of introduction of genetic lineages followed by their elimination, but also followed by introductions of new genetic lineages through transmission from outside the continent.

On a subnational basis a similar pattern persists. Clonal complexes identified using multiple locus variable-number tandem repeat analysis or WGS-defined genetic lineages move through an area and then die out [49]. This suggests that large areas of a cholera-endemic country eliminate cholera but then it is reintroduced from a neighboring area. An example is that of Tanzania in which different clonal complexes were detected as they moved through different parts of the country, some of which overlapped and one of which moved on to cause the recent outbreaks in Zanzibar [52]. A second example from Lusaka, Zambia identified 3 successive outbreaks in 2009, 2016, and 2017 each of which were caused by genetically distinct *V. cholerae* O1 that were more closely related to isolates from Tanzania or Uganda than to isolates from the other Zambian outbreaks [50]. The characterization of cholera, not just as an infection caused by *V. cholerae* O1 but rather as a specific genetic lineage of *V. cholerae* O1, illustrates that genetic lineages spread through a region within and between countries, and then die out. These findings suggest that cholera lineages appear to have been repeatedly eliminated from many areas of Africa, but then new or evolved lineages are introduced from outside the area, leading to subsequent outbreaks.

A concept that has intrigued cholera epidemiologists is the potential for cholera to reside in an environmental reservoir and then to emerge, based on suitable climatic conditions, to begin spreading from person to person. Our studies did not identify environmental, culturable *V. cholerae* O1 in either Cameroon or Uganda and the molecular data suggest that the outbreaks were caused by person-to-person spread (through fecal-oral transmission) leading to spread through an area, likely by movement of people, rather than emerging from an environmental reservoir. If *V. cholerae* was to emerge from an environmental reservoir, it would likely be of the same genetic lineage as the previous outbreak in that area. Instead, isolates generally are genetically closely related to isolates from other locations and then evolve new variation during the outbreak, as expected [53].

### Implications for the Cholera Roadmap

The GTFCC hopes to eliminate cholera from *>* 20 countries by 2030. Our studies suggest that this goal is obtainable because cholera lineages have repeatedly been eliminated from many countries and from many districts within these countries over the years. The major problem for cholera control is stopping reintroduction of cholera from outside an area. The same interventions identified in the roadmap [47] , including early identification and control of outbreaks, use of OCV, and improvement of WASH in hotspots, are still suitable for preventing transmission. However, increased emphasis is needed for broad-based surveillance to identify outbreaks at the earliest stage to prevent these outbreaks from spreading to new areas. The wide-scale use of RDT and a rapid reporting system will greatly facilitate the type of comprehensive and intensive surveillance that is required [54, 55]. Microbial culture is still needed, especially for determining antimicrobial sensitivity, but RDTs should be widely available at the district or ward level so that cases can be detected very early in an outbreak and an effective response can rapidly be mounted. Waiting for a culture result, which may take days or weeks [56], before declaring an outbreak may delay a rapid response that is needed. RDTs can be used to declare outbreaks quickly, especially if more than 1 patient is found to be positive. Preliminary studies suggest that the RDTs can also be saved in a plastic bag, sent to a laboratory, and the DNA from the dipstick can be extracted to detect *V. cholerae* using polymerase chain reaction (PCR). The DNA from the RDT may also be used for molecular characterization of the *Vibrio,* providing even more epidemiological information about disease transmission.

While vaccination of hotspot areas remains a key strategy, its use should also focus on routes of transmission, as suggested for Uganda [57] and Burundi [46], which focus on persons, including refugees, arriving from neighboring countries where cholera is common. Specific interventions for migrants must, however, be cognizant of the need to avoid stigma, yet still be effective.

Because cholera is transmitted by people when traveling, reintroduction needs to be considered as a cross-national border as well as a cross-district issue. Current methods for detecting hotspots focus on the district as the unit of analysis; however, within districts, microhotspots may better define outbreaks at the ward level and will provide a critical understanding when attempting to interrupt transmission (Ngwa, unpublished).

The definition of elimination may also need to be adjusted for susceptible countries that continue to be at risk. A country without cholera for 3 consecutive years may still be at high risk if it borders countries with continued transmission.

Other factors defined in Bangladesh need to be reexamined for Africa. For example, family studies, which revealed high rates of mild and asymptomatic infection in Bangladesh, need to be undertaken for countries in Africa. These mild infections in Bangladesh may be related to preexisting immunity and a higher proportion of infections in Africa might be severe because of lack of this immunity. Biological risk factors, such as hypochlorhydria and blood group, have not been studied in Africa. Initially, there was concern that OCV may be less effective in Africa compared to Bangladesh because the population had less natural exposure; however, studies have found the vaccine to be equally effective in Africa [27].

A summary of the epidemiological observations from Africa are shown in the right-hand column of Table 1 and are compared to those from the Ganges Delta. While our findings are based on studies from several African countries, they should not be applied to the DRC and perhaps not to Mozambique. DRC reports very high numbers of cases consistently, and so cholera is clearly endemic here, which seems unique among African countries. Similarly, the environmental conditions of the rivers and estuaries in Mozambique are more like Bangladesh and might facilitate persistence of *Vibrio* in this country. In Mozambique, genetically identical isolates of *V. cholerae* were collected 8 years apart with minimal evidence of clinical cases and no outbreaks during the intervening period [58].

The evidence seems to favor the hypothesis that cholera outbreaks are caused by reintroduction of *V. cholerae* O1 into an area rather than emerging from an environmental reservoir; however, it should be clear that we have not ruled out the possibility of an environmental reservoir in some areas of Africa. Studies to identify such a reservoir are needed but, with the possible exceptions of DRC and Mozambique, this seems unlikely. Even if cholera does not have an environmental reservoir, the association of cholera with season, temperature, rainfall, and flooding suggests an important role for climate in cholera’s transmission [59]. Whether this is a direct effect of *Vibrio* behavior and survival under different climatic and environmental conditions or results from changing behaviors during different seasons remains to be studied.

In summary, the epidemiology of cholera in most Africa countries is characterized by repeated outbreaks, most of which are relatively brief. These outbreaks result from the introduction of specific genetic lineages of *V. cholerae* into an area, following which cholera seems to disappear for a time until another outbreak occurs. The sporadic, inconsistent patterns of cholera outbreaks in Africa suggest that current estimates of disease burden overestimate the true numbers. An adjustment in the estimated disease burden should lead to a revision on cost effectiveness of various interventions. If cholera primarily spreads rather than emerges from the environment, this should lead to even more resources for early detection, reporting, and responding to outbreaks, including intervening with vaccine and WASH strategies to prevent its spread. There may be situations where a case area targeted interventions strategy, using vaccinations and intensive WASH in the neighborhood around the cases, will be appropriate [60, 61]. The wide-scale use of RDT at the ward level to rapidly detect outbreaks will facilitate the rapid response that is needed. Also, the routine inclusion of molecular characterization of outbreaks using DNA from isolates, filter paper, or RDTs will help understanding of the movement of specific genetic lineages within and between countries and help to refine interventions.

With many outbreaks that occur in Africa, we must assume that *V. cholerae* is often carried to areas outside the immediate outbreak zone; however, this does not always lead to a outbreak in the new area. Considerable effort is still needed to understand why, in the same country and even the same region of the country, some districts are rarely affected while others experience outbreaks, and to explore innovative methods to clarify the relationships between human factors, environment, climate, demography, and *Vibrio* biology that lead to the initiation, as well as the collapse, of an outbreak, and the factors that lead to the dying out of a specific *Vibrio* lineage.

## Notes

***Financial support.*** This work was supported in part by the Bill and Melinda Gates Foundation (grant number OPP1148763); and the National Institute of Allergy and Infectious Disease (grant number 5R01AI123422).

***Potential conflicts of interest.*** All authors: No reported conflicts of interest. All authors have submitted the ICMJE Form for Disclosure of Potential Conflicts of Interest. Conflicts that the editors consider relevant to the content of the manuscript have been disclosed.

Presented in part: 15th Asian Conference on Diarrhoeal Disease and Nutrition, Dhaka, Bangladesh, 29 January 2020.
